# Effects of Acute Phase Intensive Physical Activity (ACTIVE-PA) Monitoring and Education for Cardiac Patients: Pilot Study of a Randomized Controlled Trial

**DOI:** 10.2196/42235

**Published:** 2023-12-20

**Authors:** Masashi Yamashita, Kentaro Kamiya, Nobuaki Hamazaki, Shota Uchida, Takumi Noda, Emi Maekawa, Junya Ako

**Affiliations:** 1 Department of Rehabilitation Sciences Graduate School of Medical Sciences Kitasato University Sagamihara Japan; 2 Division of Research ARCE Inc Sagamihara Japan; 3 Department of Rehabilitation Kitasato University School of Allied Health Sciences Sagamihara Japan; 4 Department of Rehabilitation Kitasato University Hospital Sagamihara Japan; 5 Japan Society for the Promotion of Science Tokyo Japan; 6 Department of Cardiovascular Medicine Kitasato University School of Medicine Sagamihara Japan

**Keywords:** physical activity, monitoring, information and communication technology, cardiovascular disease, cardiovascular, cardiology, exercise, RCT, randomized, cardiac rehabilitation, fitness, accelerometer, physiotherapy, hospitalized, hospitalization, in-patient

## Abstract

**Background:**

Although physical activity (PA) decreases dramatically during hospitalization, an effective intervention method has not yet been established for this issue. We recently developed a multiperson PA monitoring system using information and communication technology (ICT) that can provide appropriate management and feedback about PA at the bedside or during rehabilitation. This ICT-based PA monitoring system can store accelerometer data on a tablet device within a few seconds and automatically display a graphical representation of activity trends during hospitalization.

**Objective:**

This randomized pilot study aims to estimate the feasibility and effect size of an educational PA intervention using our ICT monitoring system for in-hospital patients undergoing cardiac rehabilitation.

**Methods:**

A total of 41 patients (median age 70 years; 24 men) undergoing inpatient cardiac rehabilitation were randomly assigned to 2 groups as follows: wearing an accelerometer only (control) and using both an accelerometer and an ICT-based PA monitoring system. Patients assigned to the ICT group were instructed to gradually increase their step counts according to their conditions. Adherence to wearing the accelerometer was defined as having enough wear records for at least 2 days to allow for adequate analysis during the lending period. An analysis of covariance was performed to compare the change in average step count during hospitalization as a primary outcome and the 6-minute walking distance at discharge.

**Results:**

The median duration of wearing the accelerometer was 4 days in the ICT group and 6 days in the control group. Adherence was 100% (n=22) in the ICT group but 83% (n=20) in the control group. The ICT group was more active (mean difference=1370 steps, 95% CI 437-2303) and had longer 6-minute walking distances (mean difference=81.6 m, 95% CI 18.1-145.2) than the control group.

**Conclusions:**

Through this study, the possibility of introducing a multiperson PA monitoring system in a hospital and promoting PA during hospitalization was demonstrated. These findings support the rationale and feasibility of a future clinical trial to test the efficacy of this educational intervention in improving the PA and physical function of in-hospital patients.

**Trial Registration:**

University Hospital Medical Information Network UMIN000043312; http://tinyurl.com/m2bw8vkz

## Introduction

With the aging population of patients with cardiovascular diseases, frailty in these patients has recently received increasing attention [1-3]. Hospitalization events in the older adult population are the greatest risk factor for developing frailty and disability [4]. In frail older patients, reduced physical activity (PA) during hospitalization is common and leads to declining activities of daily living and adverse events, such as death or institutionalization [5]. Current cardiac rehabilitation guidelines suggest that after early release from bed, the amount of activity should be increased according to the patient’s condition [6], and using PA monitoring devices reduces adverse events in patients undergoing cardiac rehabilitation [7]. Thus, preserving PA and physical function in acute care institutions is a top priority, even for short hospitalizations.

Hospitalized patients spend most of their time lying in bed and are almost inactive [8], especially patients with frailty [9]. Despite the many reports on the importance of maintaining PA [4] and early rehabilitation [10,11], the effectiveness of interventions focusing on PA during hospitalization has not been sufficiently investigated [12,13]. Therefore, effective interventions designed to address the problem of low PA in hospitals need to be developed.

Although conventional PA monitoring can evaluate and intervene in the PA of an individual using wearable devices, [14] like pedometers, smartwatches, or smartphones for outpatients [15] and community dwellers [16], it is not easy to monitor and manage individual PAs simultaneously and in a unified manner using tools, such as electrocardiogram monitors, in a hospital with many patients. In acute care settings for patients with cardiovascular disease in particular, interventions to promote PA have been observed [17], but information and communication technology (ICT)–based PA management has not yet been established. To address this problem, we developed an ICT-based multiperson PA monitoring system that can automatically aggregate quantitative information on PA, which can then be used to provide appropriate management and personalized feedback. This randomized pilot study was conducted to estimate the feasibility and effect size of an educational PA intervention using our ICT-based PA monitoring system for in-hospital rehabilitation of patients with cardiac disease with stable pathology and the ability to leave the bed.

## Methods

### Study Design and Patient’s Randomization

The Acute Phase Intensive Physical Activity Monitoring (ACTIVE-PA-Monitoring) trial was designed as a single-center, randomized, prospective, open-label, controlled pilot trial (with 2 parallel groups and outcomes blinded). This study is reported in accordance with the Consolidated Standards of Reporting Trials (CONSORT) checklist (Multimedia Appendix 1).

**Figure 1:**
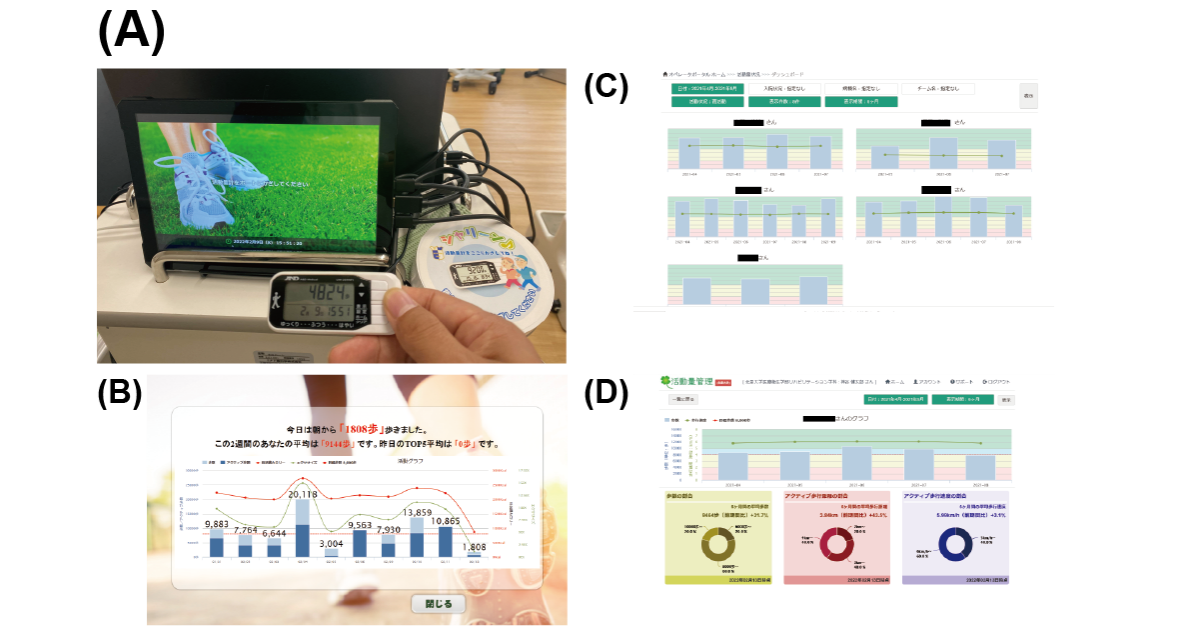
An information and communication technology–based multiperson physical activity monitoring system and the accelerometer used in this study. (A) Triaxial accelerometers and a multipatient physical activity monitoring system were used in this study. The display says, “Please hold the accelerometer to the port.” The accelerometer weighs 25 g and is 33.0 mm high, 73.8 mm wide, and 10.8 mm thick. (B) Results screen viewable by the patient. (C) A screen for medical staff to manage information from multiple patients. (D) A part of the view used to provide feedback to the patient.

Written informed consent was obtained from each patient when they could walk independently in the ward during inpatient cardiac rehabilitation between February 2021 and June 2021. All patients were 18 years or older. Patients who matched the exclusion criteria described in Table S1 of Multimedia Appendix 2 were not approached. After consent was obtained, all patients were given a small triaxial accelerometer (UW-204NFC by A&D; Figure 1). During the trial, the participants were instructed to wear the device whenever they left their beds. Checking the number of steps displayed on the accelerometer was not restricted. The measurement accuracy of this accelerometer in patients undergoing cardiac rehabilitation for approximately 5 minutes was less than 10% at various gait speeds (0.69-1.46 m/s), and the intraclass correlation coefficient between the actual and accelerometer step counts was 0.951 (Table S2 of Multimedia Appendix 2; unpublished data), which is considered acceptable compared with devices used in previous studies [18,19]. Patients were then randomly assigned to 1 of 2 groups as follows: a group receiving educational intervention using the ICT-based PA monitoring system during the study period (ICT group) or a group that wore an accelerometer only (control group). The patient’s age (younger than 75 years or 75 years or older) and 10 m gait speed (<0.8 m/s or ≥0.8 m/s) at the time of consent were used as allocation factors when assigning patients to groups using a web-based block randomization system. The researchers enrolled patients, obtained informed consent, and assigned them to groups (MY, KK, SU, and TN). The assignment results were shared only with the patients, not with the medical staff.

### Ethical Considerations

The study was conducted with patients admitted to the Cardiovascular Center of Kitasato University Hospital after the study protocol was approved by the Ethics Committee of Kitasato University Hospital (C19-298) and published on the University Hospital Medical Information Network (UMIN-CTR, unique identifier: UMIN000043312). This study was conducted entirely in adherence to the Declaration of Helsinki and Ethical Guidelines for Medical and Health Research Involving Human Subjects, promoted by the Ministry of Health, Labor, and Welfare in Japan. All patients included in this study obtained written informed consent about this study information. The content of the informed consent included that the data would be deidentified before analysis, that no honorarium would be given, and that the free will to drop out of the study would be respected.

### Educational Intervention Using the Monitoring System

We used our newly developed ICT-based PA monitoring system synchronized with a small triaxial accelerometer (Figure 1 and Multimedia Appendix 3). The PA monitoring system was set up at the entrance of the rehabilitation room in the hospital. Patients allocated to the ICT group were instructed to use this monitoring system daily, where possible, on each weekday when rehabilitation was performed. When the patient holds up the accelerometer to the system for a few seconds, a graph shows trends in activity throughout the hospital stay (Figure 1B). The amount of activity can be identified by the color of the total number of steps and the number of steps taken at moderate or greater intensity. The records stored in the monitoring system were synchronized with a tablet, which was used by the physical therapists in charge of feedback (MY, KK, SU, and TN). The physical therapists provided feedback using the tablet screen (Figure 1C and D). The patients were instructed to gradually increase the number of steps according to their condition and eventually aim for 5000 steps for the duration of their hospital stay, based on a previous study [20,21]. If the patient’s step count was less than 2000, patients were instructed to aim for 2000 steps first. No detailed protocol was defined for the frequency or length of feedback.

Patients assigned to the control group were also instructed to gradually increase their amount of PA based on the guidelines of the Japan Circulation Society [6] and to monitor their PA with their accelerometer.

### Rehabilitation Program

In this study, all patients performed a multidomain rehabilitation intervention involving supervised rehabilitation therapy to recover lower limb strength, standing balance, multimobility, and exercise tolerance through targeted exercises based on the guidelines of the Japanese Circulation Society [6]. The therapy consisted of a gym-based exercise training program with 5 minutes of stretching, balance training, and resistance training using the patient’s body weight, and 20 to 40 minutes of aerobic training using a cycling ergometer or treadmill, including warm-up and cool-down periods. The exercise intensity in both types of training was prescribed at a perceived exertion rate of 11-13 on the Borg rating of perceived exertion scale (range=6-20). The exercise intensity was increased progressively in each session. Patients participated in the rehabilitation program for 1 hour daily, 5 days per week during hospitalization, and no adverse symptoms or events were reported.

### Outcomes

The feasibility to be verified in this study is whether or not the system can be introduced and whether feedback using the system can promote an increase in the PA. Therefore, the primary outcome of this study was the average step count taken during the last 7 days of hospitalization (average steps). If the duration of hospitalization after obtaining consent was less than 7 days, the average steps until discharge were used, excluding the date on which the consent was obtained. The secondary outcomes were the number of steps on the day before discharge (steps at discharge), the change in PA between the number of steps the day after obtaining consent and average steps (Δ average step), and the average number of steps taken at moderate or greater intensity during the last 7 days of hospitalization (average active steps). Moderate or greater intensity activity was defined as 3 metabolic equivalents or more [22]. Moreover, the 6-minute walk distance (6MWD) at discharge, change in 10 m comfortable gait speed during hospitalization (Δ 10 m comfortable gait speed), and change in short physical performance battery (Δ SPPB) were secondary outcomes of physical function indicators related to PA. The measurement of 6MWD was performed by rehabilitation staff according to the American Thoracic Society guidelines [23]. Patients were instructed to try to walk as far as possible during the 6-minute period, and the results were recorded in meters. Comfortable gait speed was measured at 10 m along a 16-m walkway. At the time of measurement, a walking aid could be used if necessary. The SPPB measurement comprised 3 items in accordance with a previous study, that is, 4-m gait speed, balance, and the timing of 5 sit-to-stands [24].

### Statistical Analysis

Only patients with complete data were used for the intention-to-treat analyses. In addition, patients who were discharged from the hospital the day after consent was obtained (including patients who were scheduled to be discharged before allocation) were excluded from the primary analyses. Continuous and categorical variables were expressed as median and IQR and n (%), respectively.

Patients who were lent an accelerometer but who could not measure their steps after release due to early discharge from the hospital were excluded from the analysis as “early discharge.”‎ Adherence to wearing the accelerometer was defined as having enough wear records for at least 2 days and that allowed for adequate analysis. For certain patients who were lent an accelerometer for a sufficient period, if the number of days of wear record was less than 2, this was defined as “insufficient wearing data” and poor adherence and so was excluded from the primary analysis because the amount of analyzable data was not reached. Then, the adherence of both groups was compared without statistical analysis.

The primary analysis was conducted after visual confirmation of the absence of abnormal distributions. Average steps were compared using analysis of covariance (ANCOVA) after adjusting for baseline age, a 4-m gait speed at the time of starting cardiac rehabilitation, the baseline step count (defined as steps taken the day after lending), the duration of wearing the accelerometer, and the duration of hospitalization. The results of the ANCOVA were expressed as an estimated value, a mean difference in the 2 groups, a 95% CI, and Cohen *d* effect size. A similar analysis was performed for the secondary outcomes as follows: average active steps, steps at discharge, Δ average steps, 6MWD, Δ comfortable gait speed, and Δ SPPB. Covariates were the same as in the primary analysis, with baseline values for each outcome (6MWD remained unadjusted due to the absence of prevalues).

All analyses were performed using R (version 4.0.2; R Foundation for Statistical Computing) and JMP (version 15.1; SAS Institute Inc), and statistical significance was defined as 2-tailed *P* value less than .05.

## Results

**Figure 2:**
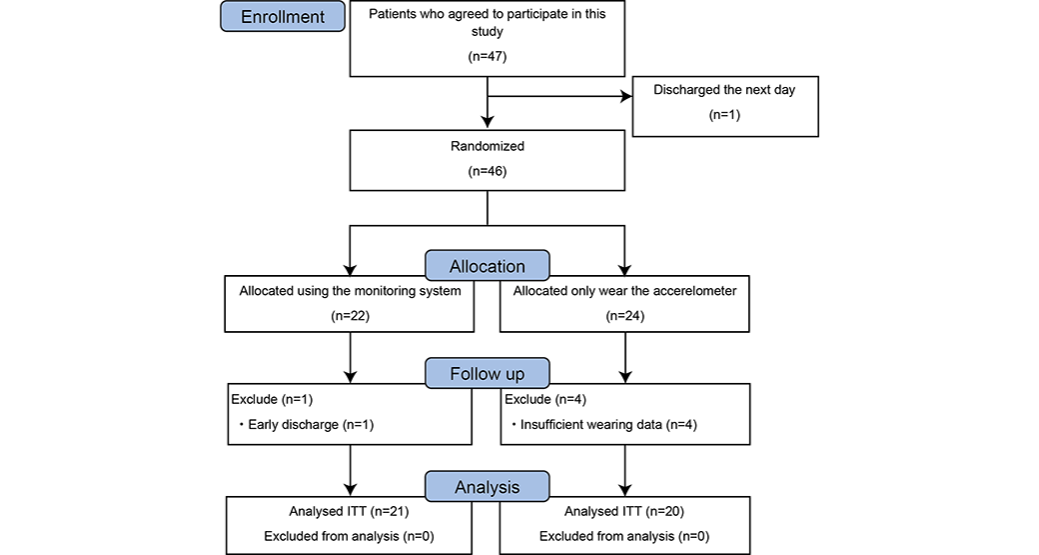
The CONSORT flow diagram in this randomized pilot trial. CONSORT: Consolidated Standards of Reporting Trials; ITT: intention to treat.

This study’s CONSORT flow diagram is shown in Figure 2. During the 5-month study period, a total of 46 patients provided informed consent and were assigned. Using random allocation, 22 patients were assigned to the ICT group, and 24 patients were assigned to the control group. Adherence to wearing an accelerometer in the ICT group was 100% (n=22). Furthermore, adequate wear records (≥2 days) were obtained from all of the 21 patients except those discharged from the hospital early. In comparison, adherence in the control group was 83% (n=20), and 4 patients had insufficient wear records (<2 days). Thus, the final analysis included 20 patients in the control group.

Comparisons of the baseline characteristics of the 2 groups are shown in Table 1. The median (IQR) age of the ICT group was 69 (60-75) years, and that of the control group was 72 (62-78) years. The proportion of patients whose primary disease was heart failure tended to be higher in the control group (ICT group: n=4, 19%; control group: n=9, 45%). The median (IQR) duration of accelerometer wear was 4 (2-7) days in the ICT group and 6 (3-9) days in the control group. Table S3 in Multimedia Appendix 2 indicates the pre- and poststudy values for each outcome. The median (IQR) number of steps at the baseline was 2764 (1872-5162) in the ICT group and 2161 (1638-3849) in the control group. The median number of active steps at baseline was 1097 (207-2397) in the ICT group and 444 (18-1615) in the control group.

**Figure 3:**
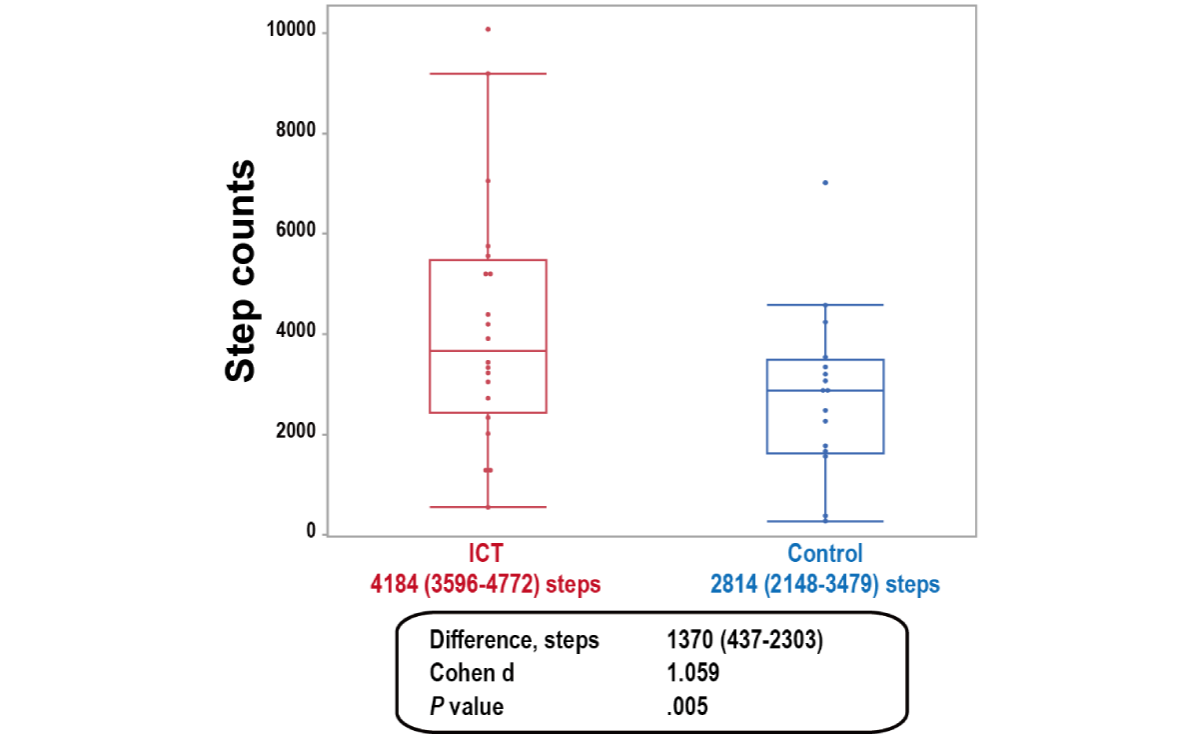
The average number of steps during hospitalization for patients using a physical activity monitoring system (red, n=21) and for patients with accelerometers only (blue, n=20) after adjustment for baseline age, a 4-m gait speed, the baseline step count, the duration of wearing the accelerometer, and the duration of hospitalization. ICT: information and communication technology.

The results of the primary ANCOVA are shown in Figure 3. The average number of steps was 2814 (95% CI 2148-3479) in the control group and 4184 (95% CI 3596-4772) in the ICT group (mean difference=1370 steps, 95% CI 437-2303; *P*=.005).

For the secondary outcomes, that is, steps at discharge (mean difference=2007 steps, 95% CI 897-3116), average active steps (mean difference=677 steps, 95% CI 25-1330), and Δ steps (mean difference=1370 steps, 95% CI 437-2303), the number of steps was significantly higher in the ICT group than in the control group, even after adjusting for covariates (Table 2). In addition, 6MWD at discharge was significantly high in the ICT group (mean difference=81.6 m, 95% CI 18.1-145.2), and a significant change was available for Δ SPPB (mean difference=0.93 points, 95% CI 0.18-1.68) compared to the control group. In terms of Δ comfortable gait speed, there was a trend toward a better ICT group (mean difference=0.17 m/s, 95% CI –0.01 to 0.35).

**Table 1:** Patient characteristics in this study (n=41).

Factor			ICT^a^ (n=21)		Control (n=20)
Age (years), median (IQR)			69 (60-75)		72 (62-78)
Gender, men, n (%)			13 (62)		11 (55)
BMI (kg/m^2^), median (IQR)			23.5 (20.4-25.2)		22.7 (20.7-25.8)
**Diagnostic category,** **n** **(%)**					
	Heart failure	4 (19)		9 (45)	
	Ischemic heart disease	7 (33)		4 (20)	
	Surgery	2 (10)		2 (10)	
	Other	8 (38)		5 (25)	
**Comorbidities,** **n** **(%)**					
	Hypertension	13 (62)		9 (45)	
	Diabetes mellitus	3 (14)		4 (20)	
	Dyslipidemia	10 (48)		5 (25)	
	Arterial fibrillation	4 (19)		8 (40)	
	Chronic kidney disease	12 (57)		12 (60)	
Albumin (mg/dL), median (IQR)			3.20 (3.00-3.80)		3.60 (3.10-3.85)
Creatinine (mg/dL), median (IQR)			0.81 (0.72-1.08)		0.92 (0.79-1.32)
Hemoglobin (mg/dL), median (IQR)			11.3 (10.1-13.6)		12.6 (10.9-13.4)
Serum sodium (mg/dL), median (IQR)			138 (137-140)		138 (136-140)
White blood cell (10^3^/μL), median (IQR)			7.70 (6.40-8.70)		6.35 (5.15-7.50)
Gait speed at the start of rehabilitation (m/s), median (IQR)			0.93 (0.64-1.11)		0.83 (0.66-0.98)
Accelerometer wearing duration (day), median (IQR)			4 (2-7)		6 (3-9)

^a^ICT: information and communication technology.

**Table 2:** Differences in secondary outcomes of physical activity and physical function between the 2 groups.

Outcome, estimate (95% CI)^a^	ICT^b^	Control	*P* value	Difference
Steps at discharge	4837 (4138 to 5537)	2831 (2039 to 3622)	.001	2007 (897 to 3116)
Average active steps	1856 (1446 to 2267)	1179 (715 to 1644)	.04	677 (25 to 1330)
Δ steps	1136 (548 to 1724)	–234 (–900 to 431)	.005	1370 (437 to 2303)
6-minute walking distance (m)	445.4 (406.5 to 484.3)	363.8 (318.8 to 408.7)	.01	81.6 (18.1 to 145.2)
Δ SPPB^c^ (points)	2.19 (1.71 to 2.66)	1.26 (0.74 to 1.77)	.02	0.93 (0.18 to 1.68)
Δ 10 m comfortable gait speed (m/s)	0.42 (0.31 to 0.54)	0.25 (0.13 to 0.37)	.06	0.17 (–0.01 to 0.35)

^a^Results shown are the estimate value (95% CI) after adjustment for baseline age, gait speed at the time of rehabilitation, the baseline value of each outcome (except for 6-minute walking distance), the duration of wearing an accelerometer, and the duration of hospitalization.

^b^ICT: information and communication technology.

^c^SPPB: short physical performance battery.

## Discussion

To the best of our knowledge, this study is the first to report on the challenges of implementing an ICT-based PA monitoring system in a hospital setting for patients undergoing cardiac rehabilitation. The findings of this study can be summarized as follows: (1) the educational intervention using the ICT-based PA monitoring system increased step counts at the time of hospital discharge more than the accelerometer alone, (2) the same trend was observed for step counts taken at moderate or greater intensity, and (3) the use of the PA monitoring system significantly improved the 6MWDs and SPPB. These findings suggest that an in-hospital educational intervention using an ICT-based PA monitoring system can increase activity during the inpatient phase and contribute to adequate performance recovery.

The greatest strength of this study is that it demonstrates the usefulness of combining accelerometers with appropriate feedback using a visual tool. When education using the ICT-based PA monitoring system was used in conjunction with the accelerometer, a difference of more than 1000 steps was observed consistently compared with the use of the accelerometer alone. The only report with a similar validation to ours is an analysis of the effectiveness of visual feedback on PA delivered via ICT devices in 93 patients admitted to a pulmonary ward [25]. The results of that report showed that ICT intervention was not effective in increasing the time spent out of bed. However, patients with independent walking abilities tend to spend more time out of bed, so they may benefit from this type of intervention. Other reports have examined the usefulness of feedback on activity in hospitalized patients who did not use ICT tools. For example, Izawa et al [17] examined self-recorded activity in 126 patients undergoing cardiac rehabilitation by dividing them into 2 groups as follows: those who recorded activity only during the acute phase and those who continued until outpatient rehabilitation. They found that step counts improved when the recording was continued until outpatient rehabilitation. In addition, Kanai et al [26] conducted a study with 55 patients with stroke using accelerometers and found that those who received feedback reported a higher increase in step counts than those who wore the accelerometer but did not receive feedback. Moreno et al [27] reported that among 58 hospitalized patients, those who received education from physical therapists took approximately 1000 steps, on average, more than those who did not receive an education. These findings support those of this study. In contrast, another previous study [28] indicated that feedback did not result in sufficient improvement of PA. Therefore, more studies are necessary to confirm the usefulness of the feedback provided by the PA monitoring system.

This study also found a significant difference in 6MWD. The 6MWD reflects exercise tolerance and is a prognostic factor for patients with cardiovascular disease. The minimum clinically important difference in 6MWD is said to be 50 m [29,30]. The Rehabilitation Therapy in Older Acute Heart Failure Patients (REHAB-HF) trial [31], the largest study in recent years that extended to outpatient rehabilitation in older and frail patients, reported an effect size of 1.5 points at 3 months for the primary outcome of SPPB and 34 m at 3 months for the secondary outcome of 6MWD. In this study, the effect size of Δ SPPB was 0.93 points, which was lower than in the previous study but significantly improved compared with the control group. In addition, the difference was more than 70 m at the time of hospital discharge, although the baseline 6MWD was not measured. Therefore, even though the intervention period was short, the combined education using the PA monitoring system and the accelerometer may have increased the amount of walking in the ward, resulting in sufficient improvement.

One possible reason for the effectiveness of this intervention may be that the ICT-based feedback system provided easy and clear access to quantitative data records. Self-monitoring is effective [17], but it poses difficulties, such as excessive burdens on the patient and issues with continuity and cognitive function. Therefore, it requires time and effort to introduce self-monitoring to all patients in a uniform way. In contrast, the ICT-based PA monitoring system introduced in this study can record data in a few seconds, giving both patients and medical professionals immediate access to the information they need. In addition, by presenting quantitative graphs, patients can see their trends, which facilitates clear feedback and goal setting. With this reduced burden on both patients and medical professionals, detailed records can be returned to a greater number of patients, leading to the identification of patients who require further intervention. This may have contributed to the excellent adherence of the monitoring group in this study.

We set a goal of 5000 steps for this study, but the average number of steps taken by the intervention group was only approximately 4000. In our experience, it is challenging to achieve improvements by only talking to patients in rehabilitation, and it has been reported that lending accelerometers to patients alone does not increase activity [32]. This may be partly explained by the living environment in the hospital ward. Interestingly, it has been shown that patients who do not have access to bedside entertainment services are likelier to walk more [33]. In addition, some patients can only walk under the supervision of nurses and other medical personnel because of frailty. To make the best use of the PA monitoring system, we believe it is important to share the activity records among multiple professions and have staff talk to patients appropriately outside the rehabilitation center, such as in the wards [34,35]. Moreover, in the future, the monitoring system could be seamlessly linked to outpatient care after discharge from the hospital, thereby maximizing the benefits of this system and promoting more improvement in PA. Moreover, maintaining PA for postdischarge patients who no longer have access to this monitoring system would be an important challenge [15].

Although this study constitutes innovative intervention research on hospitalized patients using ICT, some limitations should be considered. First, this was a single-center pilot study, and it did not have a large enough sample size. Based on the effect size results obtained by this study, to verify the effectiveness of the treatment, a proper design based on a calculated sufficient sample size is required. Second, the period between the loan of the accelerometers and discharge from the hospital was short, and it may not have been sufficient to verify the effect of the intervention. Third, the validation was conducted in an acute care hospital, and the threshold for determining good adherence may have been low due to the short length of stay. Finally, the secondary outcome of this study, 6MWD, was measured only once. Usually, 2 measurements are recommended because of the high variability associated with this test. Therefore, caution should be exercised when interpreting the results.

### Conclusions

Combining the wearing of an accelerometer with an educational PA intervention using an ICT-based portable multipatient PA monitoring system showed potential for increasing activity and exercise tolerance in hospitalized patients undergoing cardiac rehabilitation. The appropriate use of a simple and articulate feedback system during hospitalization periods could help patients reach the necessary amount of activity during hospitalization. Our findings support the rationale and feasibility of a future clinical trial to test the efficacy of the educational intervention using a multipatient PA monitoring system in improving the PA and physical function of in-hospital patients. We hope that further validation will confirm the usefulness of a PA monitoring system, such as that used in this study.

## Appendix

Multimedia Appendix 1 CONSORT-eHEALTH checklist (V 1.6.1).

Multimedia Appendix 2 Materials summarizing the exclusion criteria and the results of the sensitivity analysis in this study.

Multimedia Appendix 3 An explanatory video of the multiperson physical activity monitoring system.

## Abbreviations

6MWD: 6-minute walking distance
ACTIVE-PA: Acute Phase Intensive Physical Activity
ANCOVA: analysis of covariance
CONSORT: Consolidated Standards of Reporting Trials
ICT: information and communication technology
PA: physical activity
REHAB-HF: rehabilitation therapy in older acute heart failure patients
SPPB: short physical performance battery

## Data Availability

The data sets generated during or analyzed during this study are not publicly available due to data protection reasons but are available from the corresponding author on reasonable request.
