# Supplementary material for: Effects of Acute Phase Intensive Physical Activity (ACTIVE-PA) Monitoring and Education for Cardiac Patients: Pilot Study of a Randomized Controlled Trial
Source: J Med Internet Res. 2023 Dec 20;25:e42235. doi: 10.2196/42235 (PMC10765285; doi:10.2196/42235)
Supplement: Multimedia Appendix 2 [file jmir_v25i1e42235_app2.doc]

| **Table S1:** Exclusion criteria for this study | |
| --- | --- |
|  | 1) Significant myocardial ischemia during low-intensity exercise |
|  | 2) Acute infective endocarditis, myocarditis, or pericarditis |
|  | 3) Severe symptomatic aortic stenosis, mitral stenosis, or hypertrophic obstructive cardiomyopathy |
|  | 4) Severe pulmonary hypertension |
|  | 5) Intracardiac thrombus |
|  | 6) Untreated life-threatening arrhythmia |
|  | 7) Patients with a circulatory assist device |
|  | 8) History of aortic dissection or presence of aortic aneurysm or aortic dissection |
|  | 9) Patients with uncontrolled diabetes |
|  | 10) Peripheral arterial disease (Fontain 3–4) |
|  | 11) Recent embolism |
|  | 12) Patients undergoing hemodialysis |
|  | 13) Patients requiring assistance with walking a month before hospitalization |
|  | 14) Dementia |
|  | 15) Pregnant or lactational woman or patients who may be pregnant |
|  | |

**Table S**2: Measurement error of step counts by accelerometers used in this study in 7 cardiac rehabilitation patients

| Subjects | A | B | C | D | E | F | G |
| --- | --- | --- | --- | --- | --- | --- | --- |
| Gait speed (m/s) | 1.46 | 0.81 | 1.12 | 0.69 | 0.74 | 0.69 | 1.20 |
| Measurement error（％） | 6.9% | 4.0% | 8.8% | 1.5% | 9.3% | 1.8% | 1.7% |

This data was measured in cardiac rehabilitation patients other than those in this study (unpublished data).

**Table S3**: Outcome values of pre- and post-intervention

|  | Pre-intervention | | |  | Post-intervention | | |
| --- | --- | --- | --- | --- | --- | --- | --- |
|  | All-case | ICT ^a^ | Control |  | All-case | ICT ^a^ | Control |
| 4m comfortable gait speed [m/s] | 0.88 [0.65-1.02] | 0.93 [0.64–1.11] | 0.83 [0.66–0.98] |  | 1.09 [0.91-1.21] | 1.11 [0.97-1.33] | 1.09 [0.80-1.17] |
| SPPB ^b^ [points] | 10 [8-12] | 10 [8-12] | 11 [8-12] |  | 12 [11-12] | 12 [12-12] | 12 [10-12] |
| 6MWD ^c^ [m] | - | - | - |  | 410 [371-485] | 436 [400-510] | 403 [277-430] |
| step counts [step] | 2,358 [1,653-4,641] | 2,764 [1,872–5,162] | 2,161 [1,638–3,849] |  | 3,324 [2,021-5,910] | 4,258 [2,193-7,419] | 2,568 [1,830-4,772] |
| active step counts [step] | 735 [39-1,732] | 1,097 [207-2,397] | 444 [18-1,615] |  | 1,384 [192-2,586] | 1,588 [632-3,207] | 897 [24-2,263] |

^a^ ICT: information and communication technology.

^b^ SPPB: short physical performance battery.

^c^ 6MWD: 6-minute walking distance.
